# Preference between medical outcomes and travel times: an analysis of liver transplantation

**DOI:** 10.1007/s00423-021-02258-x

**Published:** 2021-07-29

**Authors:** Jasper Richard Burkamp, Stefanie Bühn, Andreas Schnitzbauer, Dawid Pieper

**Affiliations:** 1 Institute for Research in Operative Medicine, Faculty of Health, School of Medicine, Witten/Herdecke University, Ostmerheimer Str. 200, Building 38, 51109 Cologne, Germany; 2 Universitätsklinikum Frankfurt, Klinik für Allgemein-, Viszeral- und Transplantationschirurgie, Frankfurt am Main, Germany

**Keywords:** Patient preference, Liver Transplantation, Mortality, 3-year survival, Travel times, Transplantation Centers

## Abstract

**Background:**

There is evidence of a volume outcome relationship for liver transplantation. In Germany, there is a minimum volume threshold of 20 transplantations per year for each center. Thresholds potentially lead to centralization of the healthcare supply, generating longer travel times.

**Objective:**

This study assessed whether patients are willing to travel longer times to transplantation centers for better outcomes (lower hospital mortality and higher 3-year survival) and identified patient characteristics influencing their choices.

**Methods:**

Participants were recruited in hospitals and via random samples at registration offices. Discrete choice experiments were used to identify trade-offs in their choices between local and regional centers. Descriptive statistics and logistic regression models were used to measure patients’ preferences and quantify potentially influencing characteristics.

**Results:**

Overall, 82.22% (in-hospital mortality) and 84.44% (3-year survival) of the participants opted to accept a longer travel time in order to receive a liver transplantation with better outcomes.

**Conclusion:**

Most participants were willing to trade shorter travel times for lower mortality risks and higher 3-year survival in cases of liver transplantation.

**Supplementary Information:**

The online version contains supplementary material available at 10.1007/s00423-021-02258-x.

## Introduction

Liver transplantation in Germany is performed in specialized centers [1]. In 2018, 21 centers actually performed liver transplantation [2]. The mean distance to a liver transplantation center was estimated to be 97.4 km or 100 min, respectively [3, 4]. In-hospital mortality after liver transplantation in 2017 was 10.09%, and 3-year survival was 76.87% [5].

There is evidence of a volume-outcome relationship in liver transplantation. Patient outcomes are better at high-volume centers [6–9]. In Germany, minimum volume thresholds for liver transplantation were established in 2006 [10]. Each center has to perform at least 20 liver transplants to be allowed to conduct this procedure the following year (exemptions allowed). This regulation implicitly promotes centralization and might result in longer patient travel times. The acceptance of longer travel times of patients undergoing surgery has already been investigated in a few studies [11]. However, none of these studies has been conducted in Germany. Thus, the generalizability of those earlier studies might be limited due to cultural and health system differences.

### Objectives

This study investigated if patients were willing to travel longer times to a transplantation center in order to be treated at a center with lower in-patient mortality and a higher 3-year survival rate when information about different outcomes is available and known by potential patients.

## Materials and methods

### Study design

We used the STROBE checklist for cross-sectional studies to guarantee adequate reporting [12].

**Fig. 1:**
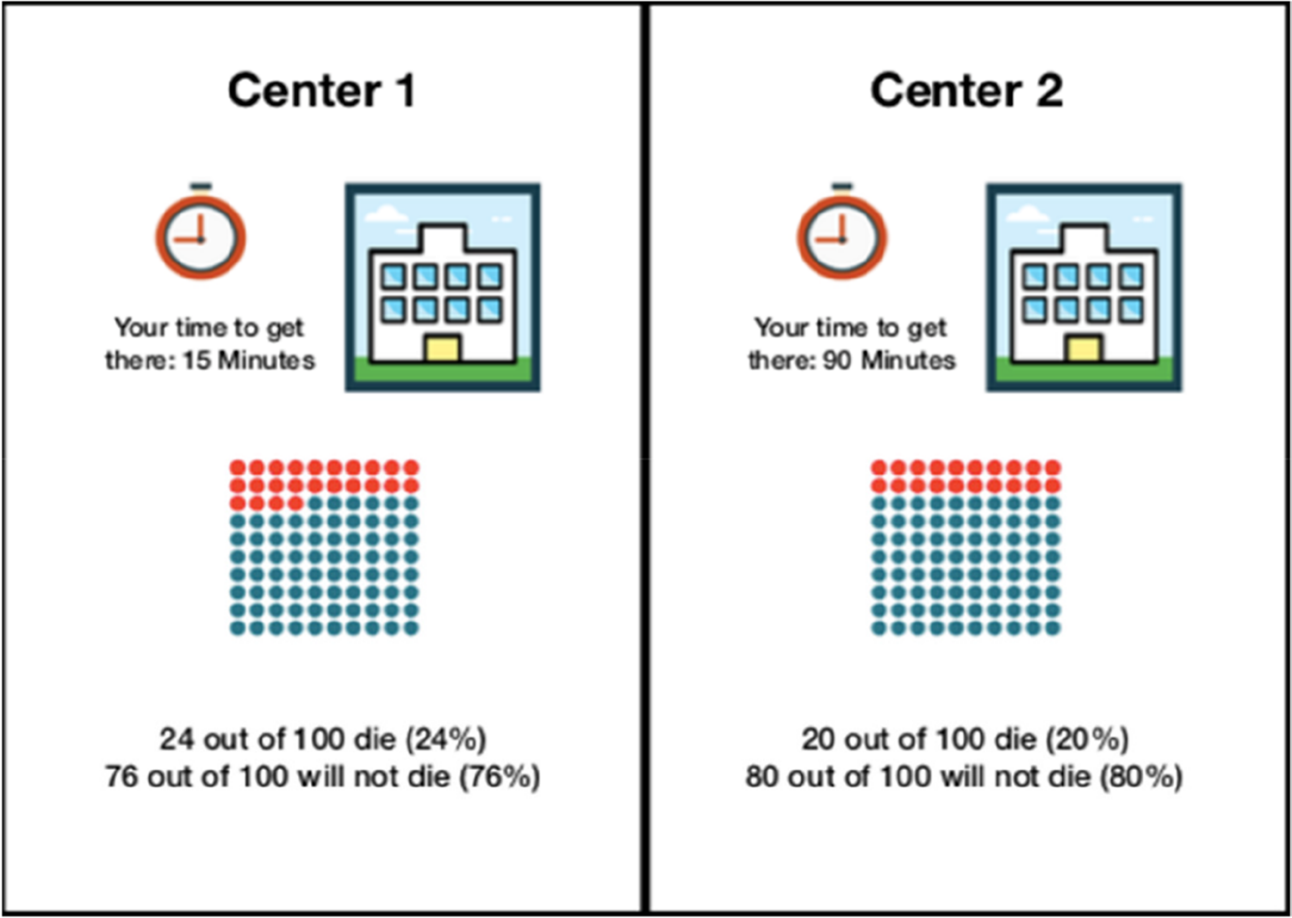
Translated visualization of the decision scenarios. Boards in the German language were used during the interviews and can be requested from the corresponding author

We analyzed decision-making behavior via a discrete choice experiment (DCE). Before the experiment, the participants received patient information about liver transplantation to ensure general understanding of the procedure and its risks. A patient information leaflet was developed together with surgeons performing liver surgery. The patient information leaflet can be found in the supporting information. To ensure that the DCE was understood, a pre-test was conducted with eight participants using the think-aloud method [13]. In the actual DCE, the participants were confronted in a hypothetical scenario with the choice of undergoing a liver transplantation at a center that could be reached by a 15-min drive (local center) and a center that could be reached within a 90-min drive (regional center) with better outcomes (lower in-hospital mortality or higher three-year survival). Risks were chosen according to their severity and probability. In 2017, the risk of in-hospital mortality after liver transplantation was 10.7%, while 75.4% of patients in Germany survived the first three years after surgery [5]. Different risks of transplantations at both centers were illustrated according to a systematic review of evidence-based risk presentation [14] (Fig. 1).

In the first decision scenario, in-hospital mortality at both the local and regional centers was equal to 24%. For participants choosing treatment at the regional center, the experiment was stopped. These participants were classified as “mortality risk-sensitive.” If the participants chose the local center, they were asked to decide in a second scenario. In the second scenario, in-hospital mortality risk at the local center remained at 24%. The risk at the regional center declined to 20%. If participants preferred treatment at the local center in the second scenario, they were asked in a third scenario. Risk of in-hospital mortality at the regional center then declined by an additional four percentage points while remaining 24% at the local center. Steps were repeated until the participant decided on the regional center.

The same procedure was repeated for the outcome 3-year survival. The initial survival rate was 50% at both centers. It increased by six percentage points at the regional center in each following iteration.

Steps of risk-reduction at the regional hospital were chosen with four and six percentage points in each iteration to properly illustrate quality improvement.

### Setting

The participants in the DCE were selected, and data were collected in 2016. The participants were recruited from an academic, tertiary hospital in Cologne, Germany, and via random samples at registration offices. Registration offices capture changes of official residences of citizens, each for a certain region in Germany with the aim to always record recent home addresses. This enabled investigation of the influence of recruitment strategy on decision-making and helped avoid selection bias. Studies show that patients in an actual medical treatment tend to differ in their decision-making related to participants that were not in an actual medical treatment [15–21].

### Participants

Inclusion criteria for participants were age between 50 and 69 years, which is the age cohort of most patients undergoing liver surgery [22]. Sufficient understanding of the German language and no mental limitations were inclusion criteria to insure understanding of the DCE.

### Variables

Choices of regional treatment in the first two scenarios were classified as mortality risk-sensitive and survival rate-sensitive. The participants in the DCE were categorized into two groups post hoc according to their choices, risk-sensitive and not-sensitive. Investigation of influences on preferences of local or regional centers was conducted via logistic regression.

### Data sources/measurement

Participants’ demographic data, characteristics, and further preferences were collected via systematic interviews before the DCE. Detailed results are provided in the supplement.

### Bias

Selection bias was reduced by recruiting the participants at the hospital and registration offices. Interviewer bias was avoided by standardized interviews and standardized information material for each participant.

### Study size

Informed by the study of Finlayson et al. [23], we originally assumed that half of the patients would choose the regional center to reduce the risks at least by 50%. To calculate a 95% confidence interval with a precision of ± 10% for a share of 50%, a sample size of at least 171 individuals was needed [11]. Therefore, the sample size was set to 180. However, this would have required eliciting the risk for each person. During the pre-test, the patients felt that this was too burdensome. Therefore, we decided to use pre-defined answers (i.e., risks) instead of eliciting them. This post hoc change results to the fact that the sample size calculation does not fully match with the analysis of the study.

Ninety participants were recruited via hospitals. Ninety participants were planned to be recruited via registration offices from different residential areas.

A total of 1,494 potential participants were identified via registration offices. The registration offices were located in rural (Vettweiß), partially urban (Bedburg), and mostly urban (Leverkusen) areas in Germany as defined by the Federal Institute for Research on Building, Urban Affairs, and Spatial Development.

### Quantitative variables

We aggregated ordinal and nominal (quantitative) data variables with multiple categories into a maximum of three categories per variable due to low cell numbers.

### Statistical methods

To select variables for multivariate models, we conducted univariate logistic regression analysis to analyze potential influence.

Variables with a p value ≤ 0.25 were taken into account for multivariate model-building [24]. Calculating regression models, all of the variables were included simultaneously (inclusion) [25]. All of the identified variables were checked for multi-correlation. In cases of multi-correlation, content-wise more sustainable variables were considered for the multivariate model. Several iterations were carried out to build the multivariate logistic model. In the first iteration, all of the variables identified via univariate models and deliberations were included. After each iteration, the variable with the highest p value was excluded. Iterations proceeded until the model quality reached an acceptable level (Nagelkerke’s R^2^ ≥ 0.200 [26]). The sensitivity analysis was conducted by classifying each participant choosing the regional center within the first three iterations as mortality risk-sensitive and survival rate-sensitive.

The statistical analysis was conducted using IBM SPSS 23.

## Results

### Participants

A total of 180 participants were recruited and participated in the DCE. Of 1,494 potential subjects identified via registration offices, 91 were interested in participating after being contacted. A total of 17 withdrew in the aftermath. Ultimately, 73 participants were recruited via registration offices. The 17 withdrawn participants were substituted by additional participants recruited at the hospital. A total of 107 participants were recruited at the hospital.

### Descriptive data

Table 1 provides an overview of the general sample characteristics. More detailed information can be found in the supplement. Overall, 52.8% of the participants were male. The average age was 59.38 years (50–69, σ = 5.517). The majority lived in mostly urban areas (62.2%). Five participants did not report their residential area, one did not report willingness to travel to the hospital, one did not report the importance of the hospital’s reputation, one did not report the importance of family or friends’ hospital recommendations, two did not report the importance of the hospital’s distance to home, and two did not report the importance of the hospital’s accessibility via public transport. Not reported/missing data were not imputed due to the small amount of missing data and therefore had a low potential impact on the results.

**Table 1:** Descriptive statistics

Sex: n (%)	
Male	95 (52.8)
Female	85 (47.2)
Age: mean (min–max, standard deviation)	59.38 (50–69; 5.517)
Living area: n (%)	
Mostly urban	112 (62.2)
Partially urban	34 (18.9)
Rural	29 (16.1)
Not reported	5 (2.8)
School degree: (%)	
Lower secondary school degree	45 (25.0)
Secondary school	46 (25.6)
High school degree	89 (49.4)
Professional training: n (%)	
No training	7 (3.9)
Practical training or other	122 (67.8)
Academic degree	51 (28.33)
Employment: n (%)	
Full time	78 (43.3)
Part time	34 (18.9)
Unemployed	68 (37.8)
Driver’s license: n (%)	
Yes	176 (97.8)
No	4 (2.2)

### Outcome data

#### Decision-making

**Fig. 2:**
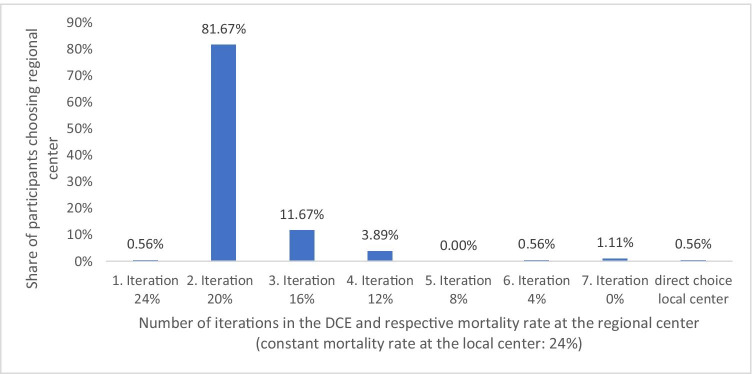
Share of participants opting for treatment at the regional center in each iteration of reducing in-hospital mortality risk at the regional center

In the initial scenario, one subject (0.56%) was willing to travel to the regional transplantation center due to the hospital’s mortality rate. In the second scenario, when the in-hospital mortality risk decreased by four percentage points at the regional center, 147 (81.67%) of the participants chose the regional center. Reducing the risk of in-hospital mortality by four percentage points at the regional center in the following iteration, another 21 subjects (11.67%) chose the regional center over the local center. By reducing the risk of in-hospital mortality at the regional center by another four percentage points, another seven subjects (3.89%) choose treatment at the regional center. The remaining two participants (1.11%) chose the regional center when the in-hospital mortality risk was 0%. One subject always chose the local center independent of the in-hospital mortality risk (Fig. 2).

**Fig. 3:**
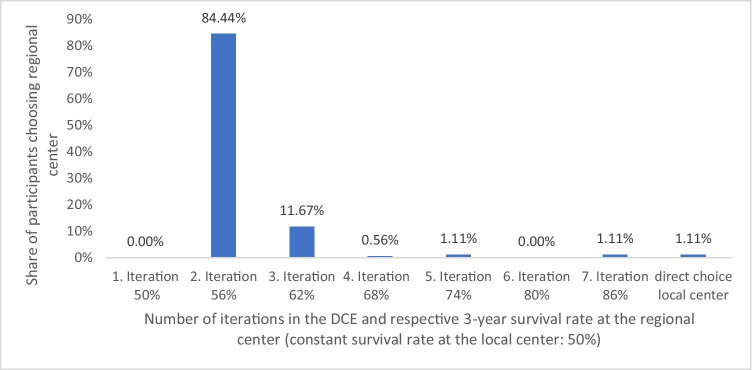
Share of participants opting for treatment at the regional center in each iteration of increasing 3-year survival rates at the regional center

No participant chose regional treatment center in the first scenario, when the chance to survive the first 3 years after transplantation at the local and regional transplantation centers was 50%. Increasing the odds of 3-year survival at the regional hospital by six percentage points to 56% convinced the majority (152 participants, 84.44%) to choose regional treatment. Increasing the odds of 3-year survival after transplantation to 62% at the regional hospital convinced another 21 (11.67%) participants to choose the regional center. The remaining two participants (1.11%) chose the regional center, when the odds of 3-year survival at the regional center were 86%. Two participants (1.11%), reported always choosing the local center (Fig. 3).

One hundred forty-eight (82.22%) of the participants were willing to travel longer times to undergo transplantation with lower in-hospital mortality risk. To be treated at a center with higher 3-year survival, 152 (84.44%) of the participants were willing to trade shorter travel times (Table 2). Regarding subgroups classified by recruitment strategy, 93 (86.91%) of the participants recruited at the hospital were willing to travel longer times to a regional transplantation center in case of a lower in-hospital mortality rate and 92 (85.98%) in case of a higher 3-year survival. Fifty-five (75.34%) of the participants recruited via registration offices chose treatment at the regional center in case of in-hospital mortality and 60 (82.19%) in case of 3-year survival (Table 2) over shorter travel times.

**Table 2:** Share of participants choosing high-quality treatment at the regional center according to the total participants and defined subgroups

	Total (n = 180)	Recruited at hospital (n = 107)	Recruited via registration offices (n = 73)	Registration office, mostly urban (n = 23)	Registration office, partially urban (n = 25)	Registration office, rural (n = 25)
Risk-sensitive mortality	82.22% (148)	86.91% (93)	75.34% (55)	78.26% (18)	72.00% (18)	76.00% (19)
Risk-sensitive three-year survival	84.44% (152)	85.98% (92)	82.19% (60)	86.95% (20)	76.00% (19)	84.00% (21)

### Main results

#### Logistic regression

The results of the univariate analysis to identify variables to include in the models are provided in the supplement.

Multivariate logistic regression analysis for the in-hospital mortality outcome revealed the significant influence of the recruitment strategy (*p* = 0.045). The participants recruited via registration office were less likely to be mortality risk-sensitive (OR = 0.424). The importance of the distance of the center had a significant influence on decision-making (*p* = 0.024). The participants stating that the importance of the center’s distance to home was “not important” were four times more likely to be mortality risk-sensitive (OR = 4.131). Employment and importance of the center equipment were included in the model without finding a significant influence but improving the model quality. The model accuracy remained poor (Nagelkerke’s R^2^ = 0.171) according to Albers et al. [26] (see Table 3).

**Table 3:** Results of the logistic regression model: in-hospital mortality

Predictor	ß	SE ß	P	Odds ratio	95% CI
Recruitment strategy Hospital Registration office	− 0.858	0.429	*0.045*	0.424	(0.183–0.983)
Employment			0.145		
Unemployed	− 0.729	0.466	0.117	0.482	(0.194–1.201)
Part time	0.308	0.666	0.644	1.361	(0.369–5.021)
Full time	Reference category				
Importance of center equipment			0.127		
Not important	0.992	0.522	0.058	0.371	(0.133–1.033)
Irrelevant	0.091	0.538	0.876	1.095	(0.349–3.436)
Important	Reference category				
Importance of distance to home			*0.024*		
Not important	1.418	0.522	*0.007*	4.131	(1.484–11.494)
Irrelevant	1.011	0.585	0.084	2.987	(0.873–8.645)
Important	Reference category				
Constant	1.43	0.545	0.009	4.177	
Test					
Overall model evaluation		χ2	df	P	
Omnibus test of model coefficients		19.309	7	0.007	
Goodness-of-fit tests					
Hosmer–Lemeshow		7.424	8	0.492	
Cox and Snell R^2^	0.103				
Nagelkerke’s R^2^	0.171				

Italicized values significance *p* < 0.05

Multivariate logistic regression analysis of the 3-year survival rate indicated a significant influence of having of a driver’s license (p = 0.033, OR = 13.432) on survival rate sensitivity. Those with a driver’s license were highly more likely to choose a center with a higher 3-year survival rate. Recruitment strategy, age, importance of the center’s reputation, importance of staff qualifications, and importance of accessibility via public transport were included in the model without finding a significant influence on the survival rate sensitivity but improving the model quality. The overall model quality remained poor (Nagelkerke’s R^2^ = 0.178, Table 4) [26].

**Table 4:** Results of logistic regression model: 3-year survival

Predictor	ß	SE ß	P	Odds ratio	95% CI
Recruitment strategy Hospital Registration office	− 0.702	0.498	0.159	0.496	(0.187–1.317)
Age	− 0.085	0.045	0.058	0.918	(0.841–1.003)
Driver’s license Yes No	2.598	1.217	*0.033*	13.432	(1.236–145.945)
Importance of center reputation			0.228		
Not important	− 0.008	0.969	0.994	0.992	(0.148–6.629)
Irrelevant	− 1.078	0.647	0.096	0.34	(0.096–1.210)
Important	Reference category				
Importance of center staff professional qualification			0.283		
Not important	− 1.723	1.084	0,112	0.179	(0.021–1.494)
Irrelevant	20.818	40,192.969	1	1,10E + 09	0
Important	Reference category				
Importance of accessibility of the center with public transport			0.074		
Not important	− 0.919	0.733	0.21	0.399	(0.095–1.678)
Irrelevant	− 1.927	0.869	*0.027*	0,146	(0.026–0.801)
Important	Reference category				
Constant	5.861	2.814	*0.037*	351.182	
Test					
Overall model evaluation		χ2	df	P	
Omnibus test of model coefficients		18.643	9	0.028	
Goodness-of-fit tests					
Hosmer–Lemeshow		7.136	7	0.415	
Cox and Snell R^2^	0.101				
Nagelkerke’s R^2^	0.178				

Italicized values significance *p *< 0.05

### Other analysis

The sensitivity analysis did not demonstrate significantly different results.

## Discussion

### Key results

The majority of the participants were willing to travel a longer time of 75 min to a transplantation center to improve their outcomes. Preferences emerged when improvements in the outcomes at the regional center were not highly distinctive in the DCE (4% and 6%, respectively).

The participants recruited at the hospital seemed to be slightly more quality sensitive than those recruited via registration offices. Out of all characteristics, only the recruitment strategy was found to be associated with decision-making. The proportion of participants recruited at the hospital categorized as quality sensitive was higher than in the subgroup of participants recruited via registration offices. These findings corresponded with the literature [15–21]. Nevertheless, statistically significant influence of the recruitment strategy on decision-making could only be found in the outcome in-hospital mortality rate and not in three-year survival. We did not identify other statistically significant patient characteristics in our multivariate logistic regression models. However, overall model quality was poor.

Analyzing two different outcomes in our study enabled comparison of the participants’ perceptions between in-hospital mortality and 3-year survival. The overall group of participants who were survival rate-sensitive was only 2.22 percentage points higher than the mortality risk-sensitive group. This gradual difference led to the assumption that the overall participants rated mortality risk and 3-year survival rates equally, leading to the assumption that there is no big difference in the rating of short- and long-term mortality outcomes. In the subgroup of participants recruited via registration offices, the difference between mortality rate sensitivity and survival rate sensitivity was more dominant. Similar differences were found in the subgroup of participants in mostly urban and rural areas, with a higher share of participants classified as survival-rate sensitive. This could be a hint, that people outside the context of a medical treatment perceive the improvement of a long-term outcome more important than the improvement of a short-term outcome. Nevertheless, the significance of these findings could not be proven due to the small sample size. Still, the impact of single decisions should be kept in mind.

### Generalizability

Comparing our findings with similar studies, differences in healthcare systems of respective countries, differences in applied methods such as recruiting strategies, execution of the DCE, investigated diseases and outcomes, and driving distances to local and regional healthcare centers have to be kept in mind.

Shalowitz et al. asked participants in a DCE to imagine being diagnosed with ovarian cancer [27]. Overall, 80% of their 60-person sample were willing to travel longer distances to have a 6% higher 5-year survival rate after initial treatment.

Chang et al. [28] used a DCE to find that 80.06% (*N* = 103) of interviewed parents were willing to travel to a regional hospital with a 3% lower mortality rate (vs 6% in a local hospital) when they imagined their child had to undergo heart surgery.

Landau et al. [29] found via a DCE, in cases of abdominal aortic aneurysm, that 91% (*N* = 67) of patients preferred treatment at a regional hospital when mortality risk at a local hospital was higher than at the regional hospital (3% vs 2%).

Finlayson et al. [23] used a DCE and found that a regional hospital was preferred by the majority (55%, *N* = 100) in cases of pancreas cancer treatment when mortality was lower than at a local hospital (3% vs 6%). Applied regression models to identify factors influencing decision-making found older age and fewer years of formal education were associated with preferences for local hospitals with worse outcomes.

In a previous study, we used the same population and methods and investigated patient preferences for elective total knee arthroplasty between driving distance and better outcomes (lower mortality risks and lower revision rates). Overall, 71.7% and 86.11%, respectively, were willing to accept longer travel times to a hospital to have lower mortality and revision rates, respectively. Lower school qualifications were identified as being associated with preferences for local treatment [30].

As summarized by Bühn et al., there was a mutual trend in all known studies analyzing the trade-off between shorter travel times and lower medical risks of surgical treatment. Participants tended to accept longer distances or travel times in order to lower surgical risks of their treatment. However, decision-making seems to be not only be determined by rational reasons such as information about outcomes and distance to the hospital. In Finlayson et al.’s study, the share of patients preferring local treatment was rather high when outcomes were worse than in the regional hospital. Even when the risk of dying in the local hospital was 100%, 10% (*n* = 10) still preferred local treatment [23].

In some studies, the proportions of participants choosing regional treatment when outcomes in local and regional hospitals were equal were rather high (Landau et al. 40%, Chang et al. 17.5%, and Shalowitz et al. 32.00%), except for Finlayson et al. (0%) and Burkamp et al. (0–1%). Bühn et al. concluded that factors other than medical outcomes and distance to hospital also may influence decision-making [11]. The regression analysis findings by Finlayson et al. and Burkamp et al. were inconsistent [23, 30]. Our recent results confirmed the general trend that better outcomes are preferred over shorter travel times. When comparing shares of patients accepting longer travel times, sample sizes should be taken into account. Most studies had small sample sizes, increasing the impact of a single decision.

### Limitations

Certain sample sizes (*n* ≥ 25) are required to analyze decision-making in relevant subgroups with logistic regression [26]. Relevant sample sizes are too small to perform further logistic regression models.

Quality improvement steps in each iteration in the DCE are rather large and might not represent real quality improvements in treatment when choosing a distant center. Nevertheless, Nijboer et al. found that the mean in-house mortality in case of liver transplantation in Germany between 2007 and 2010 was 17.6% with a range between 0 and 71.4%. The mean 3-year survival was reported with a mean of 66.0% and a range from 0 to 100% [31]. The wide range in outcomes confirms significant improvement in outcomes are possible when the transplantation center can be chosen.

Travel times were selected because the study population also underwent a DCE for outcomes of elective total knee arthroplasty [30]. Total knee arthroplasty is a far more common procedure than liver transplantation. A hospital performing total knee arthroplasty can be reached by shorter driving times. The time setting chosen represents a compromise in order to enable analysis of both issues with one survey and dataset. Further, since the participants are not actual patients that will undergo a liver transplantation, our results might not represent the decision-making of actual patients for liver transplantation.

In Germany, minimum volume thresholds did not promote further centralization of the distribution of liver transplantation centers [32]. Another limitation is that most participants lived in urban areas, therefore generalizability of the answers, e.g., for people in rural areas is limited. Because of drop out we recruited 107 instead of 90 participants in the hospital. The hospital was in an urban area. Eighty-nine (83.2%) of the participants recruited in the hospital lived in an urban area. Because of that overall the most participants (62.2%) lived in urban areas.

Our results showed that the majority of the participants were willing to trade short travel times for better outcomes. However, it remains uncertain how supply structure changes when thresholds are raised, and a travel time of more than 100 min becomes necessary. It is unclear if a travel time of more than 100 min to a center with better outcomes is tolerated in the same expression. Chang et al.’s findings promoted a lower preference for better treatment outcomes when the travel time to the regional center doubled [28]. This implies that better outcomes are not preferred unconditionally over travel times. Regarding the performance of transplantation centers above and below the minimum volume thresholds, Nimptsch et al. found that differences in mortality rates could not be affirmed [33]. Using linear regression, Nijboer et al. found that a higher 1-year overall survival correlated with a higher number of transplantations. They could not affirm this for in-house mortality and 3-year overall survival [31]. Based on the mixed findings and lack of further studies, it is uncertain if outcomes such as mortality rate and 3-year survival improve when centralization is extended by raising the minimum volume threshold.

## Conclusion

### Interpretation

The quality of healthcare providers plays a major role when patients have the opportunity to choose a treatment center. Information or knowledge about differences in quality in treatment is not always available [34, 35].

An important feature of German healthcare is patient suffrage for medical service suppliers, which empowers patients to select their physician for treatment [34]. To enable informed decision-making, potential differences in quality of high- and low-volume transplantation should be clarified. Quality reports are not frequently available or used [34]. It remains unclear if patients are aware of potential differences in the risks, they are exposed to in different transplantation centers and take this information into account when it comes to decision-making.

If information about differences in risks is available and known, centers with lower in-hospital mortality and higher 3-year survival rates are preferred over shorter travel times to healthcare providers. We show, that, in case of liver transplantation, people might be willing to accept a more centralized healthcare provision while accepting longer travel times. Using minimum-volume thresholds as an instrument for centralization of healthcare, it should be kept in mind that travel times to health care providers cannot be extended without limit. Undersupply of rural areas has to be avoided.

Ambulant structures and organized patient transport could be established to enable reasonable access to health services with better outcomes, independently of individual area of living. Furthermore, it should be tracked scientifically, if centralization of procedures, does lead to improvement of outcomes.

In the discussion of minimum volume thresholds in Germany, our findings demonstrate the necessity of measuring the quality of transplantation centers expressed via risk probabilities in a standardized and comprehensive way and make information available for patients. When it comes to modifying minimum volume thresholds by decision-makers, it is necessary to consider patient preference for the eligible procedure.

Comparing our findings to other studies showed that preferences between procedures and outcomes can differ. This restricts generalization of results on a broad range of procedures. Different procedures need to be analyzed separately using standardized methods to enable proper comparison. Future studies should analyze more than one outcome. When choosing outcomes, differences in understanding the risks should be known [36]. Different ratings of importance of the two outcomes we analyzed cannot be clarified by our findings due to the small sample sizes. Sample sizes should be sufficient to enable a more precise analysis of relevant subgroups. Recruitment strategies should also be taken into account as potential bias for the perception and rating of outcomes.

## Supplementary Information

Below is the link to the electronic supplementary material.

Supplementary file1 (DOCX 87 KB)

## Abbreviations

DCE: Discrete choice experiment
OR: Odds ratio
