# Supplementary material for: Preference between medical outcomes and travel times: an analysis of liver transplantation
Source: Langenbecks Arch Surg. 2021 Jul 29;407(2):707–16. doi: 10.1007/s00423-021-02258-x (PMC8933375; doi:10.1007/s00423-021-02258-x)
Supplement: Supplementary file 1 — Supplementary file1 (DOCX 87 KB) [file 423_2021_2258_MOESM1_ESM.docx]

Article title:

Preference between Medical Outcomes and Travel Times: An Analysis of Liver Transplantation

Journal Name:

Langenbeck's Archives of Surgery

Authors names and affilations:

Jasper Burkamp

Institute for Research in Operative Medicine, Faculty of Health, School of Medicine, Witten/Herdecke University

Mrs. Stefanie Bühn

Institute for Research in Operative Medicine, Faculty of Health, School of Medicine, Witten/Herdecke University

Prof. Andreas Schnitzbauer

Universitätsklinikum Frankfurt, Klinik für Allgemein-, Viszeral- und Transplantationschirurgie

Dr. Dawid Pieper

Institute for Research in Operative Medicine, Faculty of Health, School of Medicine, Witten/Herdecke University

E-mail corresponding author:

jasperburkamp@gmx.de

Supporting Information

|  | **N=180 (Hospital; n=107, Mostly urban: n=23, Partially urban: n=25, Rural: n=25** | **Hospital (n=107)** | **Registration office (n=73)** | | |
| --- | --- | --- | --- | --- | --- |
|  |  |  | **Mostly urban (n=23)** | **Partially urban (n=25)** | **Rural (n=25)** |
| **Sex: n (%)** |  |  |  |  |  |
| Male | 95 (52.8) | 57 (53.3) | 13 (56.5) | 14 (56.0) | 11 (44.0) |
| Female | 85 (47.2) | 50 (46.7) | 10 (43.5) | 11 (44.0) | 14 (56.0) |
| **Age: mean (min-max, standard deviation)** | 59.38 (50-69; 5.517) | 59.87 (50-69; 5.615) | 58.61 (52-69; 5.341) | 59.76 (51-68; 4.711) | 57.6 (50-69; 5.859) |
| **Relationship: n (%)** |  |  |  |  |  |
| In a relationship | 130 (72.2) | 74 (69.1) | 16 (69.5) | 21 (84.0) | 19 (76.0) |
| Not in a relationship | 50 (27.7) | 33 (30.8) | 7 (30.4) | 4 (16.0) | 6 (24.0) |
| **Nationality: n (%)** |  |  |  |  |  |
| German | 178 (98.9) | 105 (98.1) | 23 (100) | 25 (100) | 25 (100) |
| Other | 2 (1.1) | 2 (1.9) | 0 (0) | 0 (0) | 0 (0) |
| **Living area: n (%)** |  |  |  |  |  |
| Mostly urban | 112 (62.2) | 89 (83.2) | 23 (100) | 0 (0) | 0 (0) |
| Partially urban | 34 (18.9) | 9 (8.4) | 0 (0) | 25 (100) | 0 (0) |
| Rural | 29 (16.1) | 4 (3.7) | 0 (0) | 0 (0) | 25 (100) |
| Not reported | 5 (2.8) | 5 (4.7) | 0 (0) | 0 (0) | 0 (0) |
| **School degree: (%)** |  |  |  |  |  |
| Lower secondary school degree | 45 (25.0) | 28 (26.2) | 4 (17.4) | 6 (24.0) | 7 (28.0) |
| Secondary school | 46 (25.6) | 26 (24.3) | 8 (34.8) | 5 (20.0) | 7 (28.0) |
| University entrance qualification | 89 (49.4) | 53 (49.5) | 11 (47.8) | 14 (56.0) | 11 (44.0) |
| **Professional training: n (%)** |  |  |  |  |  |
| No training | 7 (3.9) | 3 (2.8) | 1 (4.35) | 1 (4.0) | 2 (8.0) |
| Practical training | 122 (67.8) | 71 (66.4) | 17 (73.91) | 16 (64.0) | 18 (72.0) |
| Academic degree | 51 (28.33) | 33 (30.8) | 5 (21.74) | 8 (32.0) | 5 (20.0) |
| **Employment: n (%)** |  |  |  |  |  |
| Full time | 78 (43.3) | 48 (44.9) | 10 (43.5) | 11 (44.0) | 9 (36.0) |
| Part time | 34 (18.9) | 16 (14.9) | 6 (26.1) | 6 (24.0) | 6 (24.0) |
| Unemployed | 68 (37.8) | 43 (40.2) | 7 (30.4) | 8 (32.0) | 10 (40.0) |
| **Hours of work/week mean (min-max; standard deviation)** | 38.01 (12-70; 13.207) | 40.34 (12-70; 12.137) | 33.78 (12-60; 14.932) | 36.28 (12-60; 12.386) | 34.25 (12-60; 15.746) |
| **Driver’s license: n (%)** |  |  |  |  |  |
| Yes | 176 (97.8) | 103 (96.3) | 23 (100) | 25 (100) | 25 (100) |
| No | 4 (2.2) | 4 (3.7) | 0 (0) | 0 (0) | 0 (0) |
| **Car owner: n (%)** |  |  |  |  |  |
| Yes | 164 (91.1) | 94 (87.9) | 20 (87) | 25 (100) | 25 (100) |
| No | 16 (8.9) | 13 (12.1) | 3 (13) | 0 (0) | 0 (0) |
| **Experience with surgery as a patient: n (%)** |  |  |  |  |  |
| Yes | 32 (17.8) | 21 (19.6) | 7 (30.4) | 3 (12.0) | 1 (4.0) |
| No | 148 (8.2) | 86 (80.4) | 16 (69.6) | 22 (88.0) | 24 (96.0) |
| **Knowing someone with experience in surgery as a patient: n (%)** |  |  |  |  |  |
| Yes | 96 (53.3) | 52 (48.6) | 12 (52.2) | 16 (64.0) | 16 (64.0) |
| No | 84 (46.7) | 55 (51.4) | 11 (47.8) | 9 (36.0) | 9 (36.0) |
| **Willingness to escort family/friends to hospital?: n (%)** |  |  |  |  |  |
| Yes | 168 (93.3) | 101 (94.4) | 21 (91.3) | 24 (96.0) | 22 (88.0) |
| No | 12 (6.7) | 6 (5.6) | 2 (8.7) | 1 (4.0) | 3 (12.0) |
| **Preferred way to travel to hospital?: n (%)** |  | | | | |
| Own car | 161 (89.4) | 96 (89.7) | 19 (82.6) | 21 (84.0) | 25 (100) |
| Other | 19 (10.55) | 11 (10.28) | 4 (17.39) | 4 (16.0) | 0 (0) |
| **Number of expected visitors within 10 days of hospital stay? mean (min-max; standard deviation): n (%)** | 11.01 (0-50; 8.756) | 11.53 (0-50; 10.233) | 19 (1-25; 6.156) | 10 (4-20; 5.109) | 11.86 (2-25; 6.374) |
| **Preferred number of visitors within a 10-day hospital-stay compared to the expected number: n (%)** |  | | | | |
| More than expected | 4 (2.2) | 2 (1.9) | 1 (4.3) | 0 (0) | 1 (4.0) |
| Same as expected | 145 (80.6) | 84 (78.5) | 20 (87) | 17 (68.0) | 24 (96.0) |
| Less than expected | 31 (17.22) | 21 (19.6) | 2 (8.7) | 8 (32.0) | 0 (0) |
| **own willingness to travel to hospital (N=179): n (%)** |  | | | | |
| low | 17 (9.4) | 9 (8.4) | 1 (4.3) | 3 (12.0) | 4 (16.0) |
| medium | 57 (31.7) | 31 (29.0) | 8 (34.8) | 6 (24.0) | 12 (48.0) |
| high | 105 (58.3) | 67 (62.6) | 13 (56.5) | 16 (64.0) | 9 (36.0) |
| **How important are the following characteristics of a transplantation center, when it comes to choose a center? 1=not important 2=less important 3=irrelevant 4=rather important 5=important Median (min-max, standard deviation)** |  | | | | |
| Center reputation | 4.28 (1-5; 1.049) | 4.44 (1-5; 0.885) | 4.48 (2-5; 0.790) | 3.52 (1-5; 1.358) | 4.16 (1-5; 1.248) |
| Center staff professional qualification | 4.73 (1-5;0.843) | 4.78 (1-5; 0.793) | 4.78 (2-5; 0.671) | 4.44 (1-5; 1.356) | 4.8 (4-5; 4.08) |
| Recommendation by family or friends | 3.31 (1-5; 1.034) | 3.31 (1-5; 1.09) | 2.91 (1-5; 0.949) | 3.32 (1-5; 1.03) | 3.68 ( 2-5; 0.748) |
| Recommendation by general practitioner | 4.03 (1-5; 1.034) | 4.07 (1-5; 0.978) | 4.22 (1-5; 1.043) | 3.6 (1-5; 1.155) | 4.08 (1-5; 1.115) |
| Center equipment | 3.69 (1-5; 1.038) | 3.73 (1-5; 1.121) | 3.65 (1-5; 1.301) | 3.52 (1-5; 1.046) | 3.72 (2-5; 0.936) |
| Center-distance to home | 2.4 (1-5; 1.264) | 2.41 (1-5; 1.291) | 2.48 (1-5; 1.123) | 2.72 (1-5; 1.173) | 2.0 (1-5; 1.323) |
| Accessibility of the center with public transport | 2.2 (1-5; 1.332) | 1.99 (1-5; 1.197) | 2.43 (1-5; 1.647) | 2.72 (1-5; 1.514) | 2.32 (1-5; 1.249) |

Descriptive Statistics of the basic population and according to the subgroups stratified according to the registration strategies.

| **Mortality** |  |  |  |
| --- | --- | --- | --- |
| **Variable** | **Odds-ratio** | **Confidence-interval** | **Significance of OR (p)** |
| Recruitment-strategy | 2.053 | 0.939-4.488 | **0.071** |
| Sex | 0.932 | 0.429-2.022 | 0.858 |
| Age | 0.971 | 0.905-1.042 | 0.42 |
| Relationship | 1.286 | 0.557-2,966 | 0.556 |
| Nationality | 0.000 |  | 0.999 |
| Living area  Rural  Partially urban  Mostly urban* | 0.783  0.919 | 0.281-2.183  0.334-2.532 | 0.895  0.640  0.871 |
| School degree  Lower secondary school degree  Secondary school  University entrance qualification* | 0.548  0.449 | 0.209-1.442  0.178-1.133 | **0.208**  0.223  0.09 |
| Professional training  No training  Practical training  Academic degree* | 0.333  0.568 | 0.053-2.115  0.216-1.492 | 0.386  0.244  0.251 |
| Employment  Unemployed  Part time  Full time* | 0.441  1.066 | 0.186-1.044  0.309-3.678 | **0.112**  0.063  0.919 |
| Hours of work per week | 0.999 | 0.957-1,043 | 0.970 |
| Driver’s license | 0.000 | - | 0.999 |
| Car owner | 1.113 | 0.297-4.164 | 0.874 |
| Knowing someone with experience in surgery as a patient | 0.783 | 0.358-1.714 | 0.541 |
| Willingness to escort family/friends to hospital | 1.655 | 0.421-6.501 | 0.471 |
| Preferred way to travel to hospital | 2.492 | 0.866-7.174 | **0.09** |
| Number of expected visitors within 10 days of hospital stay | 1.001 | 0.956-1.048 | 0.978 |
| Preferred number of visitors within a 10-day hospital-stay compared to the expected number  More than expected  Same as expected  Less than expected* | 1.143  1.754 | 0.102-12.784  0.175-17.608 | 0.626  0.914  0.633 |
| Own willingness to travel to center  Not important  Irrelevant  Important* | 0.396  0.845 | 0.121-1.292  0.356-2.008 | 0.307  0.125  0.703 |
| Importance of center reputation  Not important  Irrelevant  Important* | 0.661  0.793 | 0.169-2.582  0.244-2.582 | 0.797  0.553  0.701 |
| Importance of center staff professional qualification  Not important  Irrelevant  Important* | 1.500  - | 0.178-12.648  - | 0.933  0.709  1.000 |
| Importance of center-recommendation by family or friends  Not important  Irrelevant  Important* | 1.138  0.969 | 0.422-3.073  0.396-2.375 | 0.953  0.798  0.946 |
| Importance of center-recommendation by gp  Not important  Irrelevant  Important* | 1.625  1.702 | 0.348-7.594  0.474-6.120 | 0.619  0.537  0.415 |
| Importance of center equipment  Not important  Irrelevant  Important* | 0.411  1.330 | 0.160-1.053  0.457-3.875 | **0.103**  0.064  0.601 |
| Importance of distance to the center  Not important  Irrelevant  Important* | 3.841  2.423 | 1.491-9.898  0.842-6.968 | **0.02**  0.005  0.101 |
| Importance of accessibility of the center with public transport  Not important  Irrelevant  Important* | 2.060  1.620 | 0.828-5.126  4.31-6.092 | 0.299  0.120  0.475 |

Results of univariate logistic regression-models checking for variables to influence the “decision for a reduced mortality” (*reference category)

| **3-year-survival** |  |  |  |
| --- | --- | --- | --- |
| **Variable** | **Odds-ratio** | **Confidence-interval** | **significance of OR (p)** |
| Recruitment-strategy | 0.761 | 0.330-1.757 | 0.522 |
| Sex | 0.732 | 0.318-1.685 | 0.463 |
| Age | 0.950 | 0.880-1.027 | **0.196** |
| Relationship | 0.566 | 0.201-1.595 | 0.282 |
| Nationality | - | - | 0.999 |
| Living area  Rural  Partially urban  Mostly urban* | 0.932  0.598 | 0.323-3.429  0.270-2.125 | 0.851  0.932  0.598 |
| School degree  Lower secondary school degree  Secondary school  University entrance qualification* | 1.668  0.703 | 0.510-5.456  0.276-1.794 | 0.402  0.398  0.461 |
| Professional training  No training  Practical training  Academic degree* | 1.116  1.127 | 0.118-10.564  0.453-2.808 | 0.967 |
| Employment  Unemployed  Part time  Full time* | 0.611  1.471 | 0.249-1.500  0.378-5.729 | 0.336  0.282  0.578 |
| Hours of work per week | 0.995 | 0.952-1.040 | 0.816 |
| Driver’s license | 6.250 | 0.840-46.495 | **0.073** |
| Car owner | 3.052 | 0.964-9.660 | **0.058** |
| Knowing someone with experience in surgery as a patient | 0.659 | 0.281-1.544 | 0.337 |
| Willingness to escort family/friends to hospital | 0.513 | 0.063-4.149 | 0.513 |
| Preferred way to travel to hospital | 1.661 | 0.505-5.465 | 0.404 |
| Number of expected visitors within 10 days of hospital stay | 0.982 | 0.939-1.027 | 0.439 |
| Preferred number of visitors within a 10-day hospital-stay compared to the expected number  More than expected  Same as expected  Less than expected* | -  - | -  - | 0.711  0.999  0.999 |
| Own willingness to travel to center  Not important  Irrelevant  Important* | 0.565  0.664 | 0.140-2.274  0.271-1.627 | 0.565  0.422  0.371 |
| Importance of center reputation  Not important  Irrelevant  Important* | 0.443  0.288 | 0.111-1.770  0.097-0.857 | **0.060**  0.249  0.025 |
| Importance of center staff professional qualification  Not important  Irrelevant  Important* | 0.263  - | 0.059-1.174  - | **0.216**  0.08  1.000 |
| Importance of center-recommendation by family or friends  Not important  Irrelevant  Important* | 1.215  1.351 | 0.426-3.463  0.501-3.639 | 0.826  0.716  0.552 |
| Importance of center-recommendation by gp  Not important  Irrelevant  Important* | 0.784  2.082 | 0.206-2.992  0.456-9.498 | 0.575  0.722  0.344 |
| Importance of center equipment  Not important  Irrelevant  Important* | 0.734  0.931 | 0.242-2.228  0.335-2.590 | 0.862  0.585  0.891 |
| Importance of distance to the center  Not important  Irrelevant  Important* | 1.852  1.820 | 0.667-5.142  0.548-6.046 | 0.464  0.237  0.328 |
| Importance of accessibility of the center with public transport  Not important  Irrelevant  Important* | 0.586  0.258 | 0.161-2.133  0.057-1.1770 | 0.173  0.418  0.079 |

Results of univariate logistic regression-models checking for variables to influence the “decision for a higher 3-year-survival” (*reference category)
